# A Novel Adaptive Signal Processing Method Based on Enhanced Empirical Wavelet Transform Technology

**DOI:** 10.3390/s18103323

**Published:** 2018-10-03

**Authors:** Huimin Zhao, Shaoyan Zuo, Ming Hou, Wei Liu, Ling Yu, Xinhua Yang, Wu Deng

**Affiliations:** 1 Software Institute, Dalian Jiaotong University, Dalian 116028, China; hm_zhao1977@126.com (H.Z.); smhhbdjtu@163.com (S.Z.); yangxh@djtu.edu.cn (X.Y.); 2 Co-innovation Center of Shandong Colleges and Universities: Future Intelligent Computing, Yantai 264005, China; 3 Traction Power State Key Laboratory of Southwest Jiaotong University, Chengdu 610031, China; 4 Liaoning Key Laboratory of Welding and Reliability of Rail Transportation Equipment, Dalian Jiaotong University, Dalian 116028, China; 5 Chuzhou Technical Supervision and Testing Center, Chuzhou 239000, China; liudayou0804@sina.com; 6 China Household Electric Appliance Research Institute, Beijing 100037, China; liuw@cheari.com (W.L.); yul@cheari.com (L.Y.); 7 Guangxi Key Lab of Multi-Source Information Mining & Security, Guangxi Normal University, Guilin 541004, China

**Keywords:** empirical wavelet transform, maximum-minimum length curve, scale space transformation, spectrum segmentation, feature extraction

## Abstract

Empirical wavelet transform (EWT) is a novel adaptive signal decomposition method, whose main shortcoming is the fact that Fourier segmentation is strongly dependent on the local maxima of the amplitudes of the Fourier spectrum. An enhanced empirical wavelet transform (MSCEWT) based on maximum-minimum length curve method is proposed to realize fault diagnosis of motor bearings. The maximum-minimum length curve method transforms the original vibration signal spectrum to scale space in order to obtain a set of minimum length curves, and find the maximum length curve value in the set of the minimum length curve values for obtaining the number of the spectrum decomposition intervals. The MSCEWT method is used to decompose the vibration signal into a series of intrinsic mode functions (IMFs), which are processed by Hilbert transform. Then the frequency of each component is extracted by power spectrum and compared with the theoretical value of motor bearing fault feature frequency in order to determine and obtain fault diagnosis result. In order to verify the effectiveness of the MSCEWT method for fault diagnosis, the actual motor bearing vibration signals are selected and the empirical mode decomposition (EMD) and ensemble empirical mode decomposition (EEMD) methods are selected for comparative analysis in here. The results show that the maximum-minimum length curve method can enhance EWT method and the MSCEWT method can solve the shortcomings of the Fourier spectrum segmentation and can effectively decompose the bearing vibration signal for obtaining less number of intrinsic mode function (IMF) components than the EMD and EEMD methods. It can effectively extract the fault feature frequency of the motor bearing and realize fault diagnosis. Therefore, the study provides a new method for fault diagnosis of rotating machinery.

## 1. Introduction

Rolling bearings are the key component of rotating machinery. Their vibration signals are generally very complex, and especially when faults occur, the vibration signals are complicated by strong background noise, and obvious non-linear and non-stationary features. The time-frequency analysis method is to describe the changed frequency spectrum of the signal with time, establishing a distribution spectrum that can simultaneously express the energy or intensity of the signal in time and frequency [1]. They can extract the contained fault features from the signal. The time-frequency analysis method has been widely applied in the field of mechanical fault diagnosis because it can provide local information about signals in the time domain and frequency domain [2,3,4,5,6,7]. The time-frequency analysis methods include Wigner-Ville distribution, wavelet transform, S transform, spectral kurtosis, Gabor transform, sparse decomposition, empirical mode decomposition (EMD), local mean decomposition (LMD), local feature scale decomposition (LCD), ensemble empirical mode decomposition (EEMD) and so on [8,9,10,11,12]. Research on wavelet transforms in fault diagnosis is mainly based on combining wavelet analysis with other signal analysis methods, diagnosis methods. EMD is an adaptive signal time-frequency processing method proposed by Huang. It can directly decompose the signal and adaptively obtain the basis function, but it suffers from endpoint effects and mode mixing problems, which limit its application in actual engineering problems [13,14,15,16,17,18]. The EEMD method can effectively solve the mode mixing phenomenon, but this method requires a large amount of computation time.

Empirical wavelet transform (EWT) is a novel signal processing method proposed by Gilles [19]. This method combines the good characteristics of the wavelet transform and EMD methods, and can extract a series of amplitude modulated-frequency modulated (AM-FM) signals from the given signal. The EWT is used for weak fault diagnosis and identification. A compact supporting Fourier spectrum can be obtained by using the AM-FM components. The adaptive wavelets capable of AM-FM components are constructed using differential methods [20]. The differential mode is used to segment the Fourier spectrum, and some filters are used for each obtained supporting feature. The EWT has good adaptability and a reliable theoretical mathematical deduction basis, and has been effectively applied to engineering practice, but in actual engineering applications, high frequency interference components often exist in the signal spectrum due to strong background noise, which makes the signal spectrum interval of EWT decomposition not be accurate enough, and the mode components after decomposition will not contain accurate fault feature information. In recent years, some experts and scholars have deeply studied and improved the EWT method, and many improved EWT methods have been proposed. Amezquita-Sanchez and Adeli [21] proposed a new adaptive multiple signal classification EWT method in order to accurately represent time-frequency results. Kedadouche et al. [22] proposed a new signal analysis method based on EWT and operational modal analysis, which is used to decompose the signal into multiple components to extract the related frequency. Zheng et al. [23] proposed an adaptive parameterless EWT to fulfill an adaptive separation of the Fourier spectrum in EWT. Hu et al. [24] proposed an enhanced EWT method based on the advantages of the waveform in order to eliminate the disadvantage of basic EWT in spectrum segmentation. Thirumala et al. [25] proposed a generalized empirical wavelet transform to estimate the time-varying PQ indices for accurate interpretation of disturbances. Merainani et al. [26] used the EWT method to develop an appropriate wavelet filter bank for the early detection and condition monitoring of faults. Shi et al. [27] proposed an enhanced EWT by developing a feasible and efficient spectrum segmentation method in order to improve the accuracy of the EWT results. Hu et al. [28] proposed an enhanced empirical wavelet transform algorithm to extract bearing fault features. Hu et al. [29] proposed multi-taper empirical wavelet transform to separate the fault features from the masking components for a wind turbine planetary gearbox under nonstationary conditions. Wang et al. [30] proposed a sparsity guided EWT method, which is used to automatically build Fourier segments for fault diagnosis. Song et al. [31] proposed an improved empirical wavelet transform with adaptive empirical mode segmentation and the merging of redundant empirical modes for fault diagnosis of roller bearings. Merainani et al. [32] proposed a new self-adaptive time-frequency analysis, called Hilbert empirical wavelet transform, for obtaining the instantaneous amplitude matrices of the vibration signals. Bhattacharyya et al. [33] enhanced the existing empirical wavelet transform by using Fourier-Besse series expansion in order to obtain an improved TF representation of non-stationary signals.

Although these improved EWT methods can improve the quality of the decomposed mode components, the spectrum segmentation mode based on the frequency domain extremum point of the EWT method does not change. In addition, when motor bearing faults occur, the fault feature is usually weak and easily influenced by interference signals. The signal has a strong non-linear and non-stationary multi-component amplitude modulation feature. Because the fault features are reflected in the complex edge band information, when the EWT method is directly used to decompose the vibration signal, it will occur that many mode components will be close to the component and it is easy to lose the fault feature signal. Therefore, an enhanced empirical wavelet transform (MSCEWT) based on maximum-minimum length curve method is proposed in this paper. The maximum-minimum length curve method transforms the spectrum of the original vibration signal to the scale space in order to obtain a set of minimum length curves, and find the maximum length curve value in the set of the minimum length curve values for obtaining the number of the spectrum decomposition intervals. The MSCEWT method is used to decompose the vibration signal to obtain a series of IMFs. The Hilbert transform is the used to process these IMF components in order to obtain the fault feature frequencies of motor bearings. The actual motor bearing vibration signals are used to verify the effectiveness of the MSCEWT method for fault diagnosis.

The rest of the paper is organized as follows: in Section 2, the empirical wavelet transform method is introduced. The empirical wavelet transform method based on scale space is introduced and analyzed in Section 3. In the Section 4, an enhanced EWT (MSCEWT) based on maximum-minimum length curve method is proposed to realize fault diagnosis of motor bearings. Experimental comparison and analyses are discussed in the Section 5. Finally, our conclusions are drawn in Section 6.

## 2. Empirical Wavelet Transform

The EWT method, proposed by Gilles, is used to establish adaptive wavelets and extract the AM-FM components [19]. In the EWT method, it is firstly used to normalize the Fourier frequency spectrum and separate it into a number of intervals, then the tightly supported orthonormal wavelet base is explicitly constructed in each interval. Thus, an important step for wavelet adaptation signals is the segmentation of the Fourier spectrum segmentation. Firstly, we must calculate the local maximum in the Fourier spectrum of the signal. Then the local maxima of the Fourier spectrum are detected and the corresponding spectrum is segmented. Lastly, a filter bank based on bands is built.

### 2.1. The Empirical Scaling Function and the EWT

**Figure 1:**
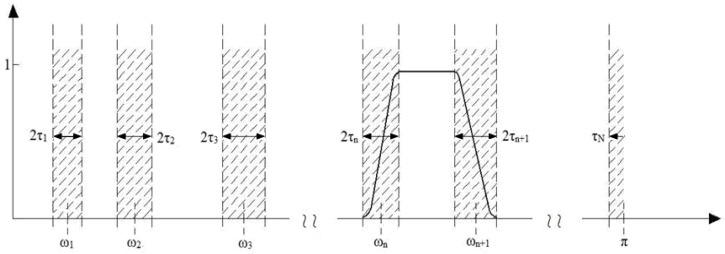
The interval division of Fourier axis.

Supposing that a signal consists of *N* components, and these components can be well separated in the Fourier spectrum. The Fourier spectrum can be divided into *N* segments, and each segment corresponds to one mode. The boundary limits between each segment is $\omega_{n}$, $\omega_{0}$ = 0 and $\omega_{N}$ = π. The interval division of the Fourier axis is shown in Figure 1. Each segment is indicated $\Lambda_{n}=\left[\omega_{n-1},\omega_{n}\right]$, then it is easy to see that $U_{n=1}^{N}\Lambda_{n}=\left[0,\pi \right]$. Centered around each $\omega_{n}$, it defines the transition phase (the gray hatched areas on Figure 1) $T_{n}$ of width $2\tau_{n}$. $\tau_{n}$ is set as $\tau_{n}=\gamma \omega_{n}$ (0<γ<1) to simplify the equation.

The empirical scaling function and wavelets are defined as follows [19]:

(1) $${\hat{\phi }}_{n}(\omega )=\{\begin{array}{cc} 1 & if\;|\omega |\leq \;(1-\gamma )\omega_{n}\\ \cos[\frac{\pi }{2}\beta (\frac{1}{2\gamma \omega_{n}}(|\omega |-(1-\gamma )\omega_{n}))] & if\;(1-\gamma )\omega_{n}\leq |\omega |\leq (1+\gamma )\omega_{n}\\ 0 & otherwise \end{array}$$

(2) $${\hat{\psi }}_{n}(\omega )=\{\begin{array}{cc} 1 & if\;(1+\gamma )\omega_{n}\leq |\omega |\leq \;(1-\gamma )\omega_{n+1}\\ \cos[\frac{\pi }{2}\beta (\frac{1}{2\gamma \omega_{n+1}}(|\omega |-(1-\gamma )\omega_{n+1}))] & if\;(1-\gamma )\omega_{n+1}\leq |\omega |\leq \;(1+\gamma )\omega_{n+1}\\ \sin[\frac{\pi }{2}\beta (\frac{\pi }{2\gamma \omega_{n}})(|\omega |-(1-\gamma )\omega_{n})] & if\;(1-\gamma )\omega_{n}\leq |\omega |\leq (1+\gamma )\omega_{n}\\ 0 & otherwise \end{array}$$

The function β(x) is an arbitrary $C^{k}[0,1]$ function such as:

(3) $$\beta (x)=\left\{ \begin{array}{ll} 0 & if\;x\leq 0\\ 1 & if\;x\geq 0 \end{array}\right.\;and\;\beta (x)+\beta (1-x)=1\;\forall \;x\in [0,1]\;$$

β(x) is represented by the following expression. The following polynomial firstly suggested by Daubechies and used by Gilles:

(4) $$\beta (x)=x^{4}(35-84x+70x^{2}-20x^{3})\;$$

Parameter γ can ensure that there is no overlap between two consecutive transition areas, so the parameter γ must meet the following condition:

(5) $$\gamma <\min_{n}(\frac{\omega_{n+1}-\omega_{n}}{\omega_{n+1}+\omega_{n}})\;$$

### 2.2. Segmentation of Fourier Spectrum

In the empirical wavelet transform, the Fourier spectrum segmentation is an important step, laying a good foundation for signal analysis. We divide the spectrum into different parts that correspond to tight support at a specific frequency. If the Fourier transform of the original signal *f*(*x*) is *f*(*ω*), *ω =* [0, *π*], and $\omega_{n}$ is defined as the interval boundary, the minimum between local maxima is used. The method of “localmaxmin” determines its boundary. Assuming that ${℧}_{n}$ is a local minimum set between $\omega_{n-1}$ and $\omega_{n}$, then:

(6) $$\;\omega_{0}=0,\;\omega_{N}=\pi ,\omega_{n}=argmin{℧}_{n}\;$$

where: 1≤n≤N−1. Any interval can be expressed as $\Lambda_{n}=\left[\omega_{n-1},\omega_{n}\right]$, and there are $U_{n=1}^{N}\Lambda_{n}=\left[0,\pi \right]$.

### 2.3. Basic Principle of EWT

From the previous section, we know how to build a tight frame set of empirical wavelets. We can define the EWT, $W_{f}^{\varepsilon }\left(n,t\right)$, in the sameway as for the classic wavelet transform. The detailed coefficients $W_{f}^{\varepsilon }(n,t)$ are given by the inner products with empirical wavelets:

(7) $$W_{f}^{\varepsilon }(n,t)=\langle f,\psi_{n}\rangle =\int f(\tau )\bar{\psi_{n}(\tau -t)}d\tau \;$$

and the approximation coefficients $W_{f}^{\varepsilon }(0,t)$ is given by the inner products with scaling function:

(8) $$W_{f}^{\varepsilon }(0,t)=\langle f,\phi_{1}\rangle =\int f(\tau )\bar{\phi_{1}(\tau -t)}d\tau \;$$

Then the reconstruction signal and empirical mode are given as follows:

(9) $$f(t)=W_{f}^{\varepsilon }(0,t)\times \varphi_{1}(t)+\sum_{n=1}^{N}W_{f}^{\varepsilon }(n,t)\times \psi_{n}(t)\;$$

(10) $$f_{0}(t)=W_{f}^{\varepsilon }(0,t)\times \varphi_{1}(t)\;$$

(11) $$f_{k}(t)=W_{f}^{\varepsilon }(k,t)\times \psi_{k}(t)\;$$

## 3. The Maximum-Minimum Length Curve Method

The EWT is an adaptive signal processing method. Based on the adaptive Fourier spectrum segmentation of the signal, a bank of wavelet filters is constructed in order to decompose the signal into a set of amplitude modulation and frequency modulation terms. Because the conventional LocalMaxmin method needs to determine the number of segmentations in advance, it is affected by human subjective factors in the process of implementation, which will result in the inaccurate interval decomposition and cannot achieve the purpose of adaptive spectrum segmentation. Gilles proposed the adaptive and adaptivereg methods to segment the spectrum, but one needs to preset the initial boundary vector when the EWT method segments the interval. Next, Gilles also proposed a new method of Fourier spectrum segmentation based on scale-space representation and K-Means clustering algorithm, which uses the scale space to achieve adaptive segmentation of the spectrum without presetting interval numbers.

Let a function f(x) be defined in the interval $\left[0,\;x_{max}\right]$ and the Gauss kernel function $g\left(x;t\right)=\frac{1}{\sqrt{2\pi t}}e^{-x^{2}/\left(2t\right)}$ is defined. The scale space representation is described as follows:

(12)  L(x,t)=g(x;t)×f(x) 

where × denotes the convolution product and denotes scale parameter.

In practical applications, the Fourier transform f(ω) of the original function and the sampled Gaussian kernel function are used to describe the scale space representation:

(13) $$\;L\left(\omega ,t\right)=\overset{+M}{\underset{n=-M}{\sum }}g\left(n;t\right)\times f\left(\omega -n\right)\;$$

When *M* is large enough, the error of Gaussian approximation is negligible. In the text, $M=C\sqrt{t}+1\;$is set, where 3≤C≤6. In order to meet the error that is small enough, the set C=6.

The scale space representation can be interpreted as the global trend of signals under different scale parameters, that is, all the scale space curves $C_{i}\left(i\in \left[1,N_{0}\right]\right)$ under different scale parameters are calculated, and then the threshold of the scale parameter is determined. For the scale space representation $L\left(\omega ,t_{0}\right)$ under the initial scale parameter ($\sqrt{t_{0}}$ = 0.5), the minimum value among the local maximum values in $L\left(\omega ,t_{0}\right)$ is obtained. When there is *t* = 0, the number of local minimum value obtained is obtained and regarded as $N_{0}$. These local minimum values are used to construct the scale space curve $C_{i}\left(i\in \left[1,N_{0}\right]\right)$. In the scale space representation plane L(ω,t), the calculated $N_{0}$ at *t* = 0 is recorded as the number of minimum length curves $L_{i}$. With the increasing of the scale *t*, the number of the local minimum values in L(ω,t) is decreasing until the number of minimums is reduced to 0. At this time, the value of the minimum length curve $L_{i}$ is calculated. The local minimum value of f(ω) at *t* = 0 is used to represent the $C_{1}$ starting of scale space curve that the first length of scale space curve is 1. The scale space curve is sequentially accumulated under different scales $\;t_{k}$($k=1,2,\ldots 2N_{0}$) in order to obtain the first minimum length curve $L_{1}$. Then the position of the local minimum value is determined at *t* = 0 in turn. From the starting points of these positions, the scale space curves are sequentially accumulated under different scales $\;t_{k}$($k=1,2,\ldots 2N_{0}$) in order to obtain the minimum length curves $L_{i}\left(i=1,2,\ldots ,N_{0}\right)$. Therefore, a proportional threshold *T* can be set from the minimum length curve $L_{i}$, that is to say all minimum length curves corresponding to the proportional threshold *T* is set in the minimum length curves $L_{i}$. That is, all obtained minimum length curves are greater than the threshold *T*, which are found out. The problem is transformed into a clustering problem, so that the clusters of the set ${\left\{L_{i}\right\}}_{i=\left[1,N_{0}\right]}$ are divided into two clusters (meaningful/non-meaningful minima). Here, the method of k-Means is used for clustering.

**Figure 2:**
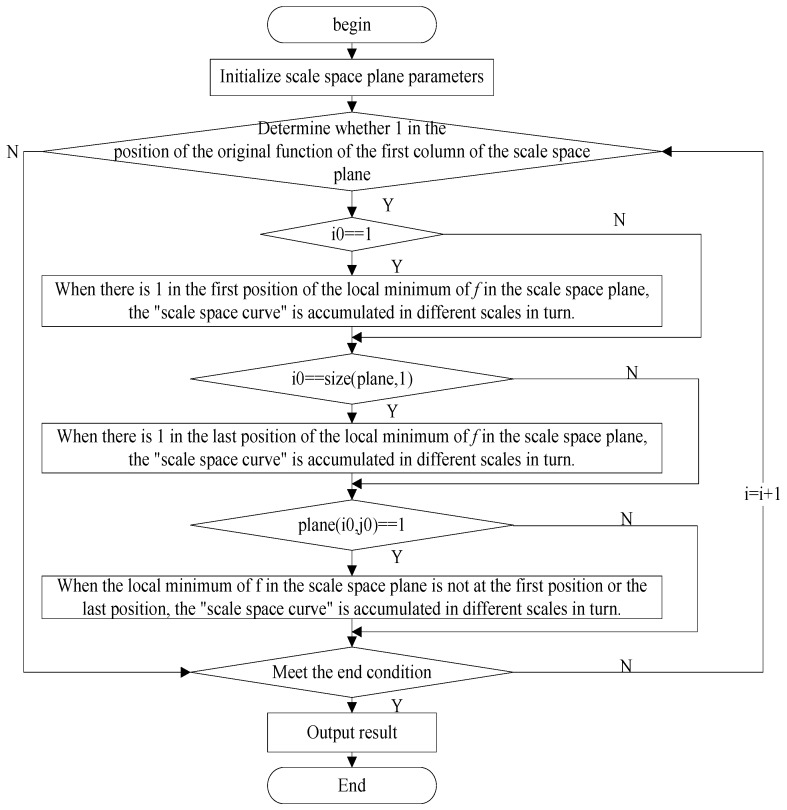
The flow of maximum-minimum length curve method.

The calculation flow of the minimum length curve is shown in Figure 2.

The calculation steps of the minimum length curve are described as follows:

*Step 1.* Initialize the scale space plane, including the first column number *i* in the scale space plane (the local minimum point of original function), and the number *ic* of the local minimum value of the original function.

*Step 2.* Determine whether the first column position in the scale space plane is 1. If the first column position is 1, Step 3 is executed. Otherwise execute Step 6.

*Step 3.* When the first position in the scale space plane exists the local minimum value of the original function, the scale space curves $C_{i}$ are sequentially accumulated under different scales in order to obtain the minimum length curves $L_{ic}$. Otherwise execute Step 4.

*Step 4.* When the last position in the scale space plane exists the local minimum value of the original function, the scale space curves $C_{i}$ are sequentially accumulated under different scales in order to obtain the minimum length curves $L_{ic}$. Otherwise execute Step 5.

*Step 5.* When the local minimum value of the original function in the scale space plane does not exist the first position or the last position, the scale space curves $C_{i}$ are sequentially accumulated under different scales in order to obtain the minimum length curves $L_{ic}$. Otherwise execute Step 7.

*Step 6.* Calculate the first column number *i* = *i* + 1 in the scale space plane.

*Step 7.* If the end condition *i* ≤ size (plane, 1) is satisfied, the minimum length curve *L*(*ic*) is output. Otherwise go to Step 6.

## 4. An Enhanced EWT (MSCEWT) Based on Maximum-Minimum Length Curve

### 4.1. The Idea of the MSCEWT Method

As a novel signal processing method, the EWT method still has a problem to extract physical frequency components from actual complex mechanical signals. In the process of Fourier spectrum segmentation of the signal, when a fine frequency interval is set, many narrowband modes are extracted, and several modes could display the same modulation information, which will result in unnecessary redundancy. In order to avoid this problem of the EWT method, an improved scale space representation (maximum-minimum length curve) method is proposed in this paper. That is an enhanced empirical wavelet transform (MSCEWT) method based on maximum-minimum length curve, which is used to decompose mechanical vibration signals in order to realize mechanical fault diagnosis. In the MSCEWT, the local minimum value in L(ω,t) is used to adaptively segment the spectrum in order to determine the boundaries, and the corresponding position of the maximum value of all the minimum length curves in the scale space representation is determined. Then the signal is decomposed into as a series of single packets with fault information, and the power spectral density is applied to the single component in order to extract the modulation information. The MSCEWT method can avoid the over decomposition phenomenon of the mode at utmost.

### 4.2. The Flow and Steps of the MSCEWT Method

**Figure 3:**
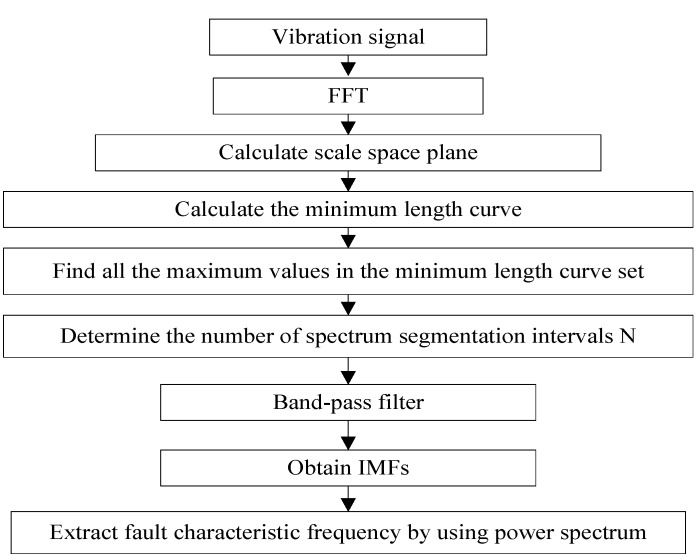
The flow of the MSCEWT method.

Firstly, the Fourier transform f(ω) of the original function and the sampled Gaussian kernel function $g\left(n;t\right)=\frac{1}{\sqrt{2\pi t}}e^{-n^{2}/\left(2t\right)}$ are convoluted in order to obtain the scale space representation. Secondly, in the calculation process, with the increasing of the scale *t*, the scale space curve $C_{i}$ is obtained. Then, the position of the local minimum is determined in turn at *t* = 0. From the starting points of these positions, the scale space curves are sequentially accumulated under different scales $\;t_{k}$($k=1,2,\ldots 2N_{0}$) in order to obtain the minimum length curves $L_{i}\left(i=1,2,\ldots ,N_{0}\right)$. The final difference between scale space representation method and maximum-minimum length curve is that the problem is converted to calculate all the maximum thresholds of the minimum length curve $L_{i}$ in the scale space representation set ${\left\{L_{i}\right\}}_{i=\left[1,N_{0}\right]}$, then the boundaries are determined by detecting the local minimum value. The number *N* of the obtained maximum threshold by calculating is defined as the number of spectrum segmentation. The flow of the MSCEWT method is shown in Figure 3. The specific steps of the MSCEWT method are described as follows:

*Step 1.* The original signal f(x) is transformed to obtain Fourier spectrum f(ω) by FFT.

*Step 2.* Execute convolution operations between f(ω) and the Gaussian kernel function g(x;t) to obtain a scale space representation, denoted as ${\left\{L_{i}\right\}}_{i=\left[1,N_{0}\right]}$.

*Step 3.* Calculatethe point of the local minimum value of f(ω) when there is *t* = 0. From the starting points of these positions, the scale space curves are sequentially accumulated under different scales $\;t_{k}$($k=1,2,\ldots 2N_{0}$) in order to obtain the minimum length curves $L_{i}\left(i=1,2,\ldots ,N_{0}\right)$.

*Step 4.* Calculate all maximum $\mathrm{values}\;max{\left\{L_{i}\right\}}_{i=\left[1,N_{0}\right]}$ of the minimum length curve $L_{i}$ in the set ${\left\{L_{i}\right\}}_{i=\left[1,N_{0}\right]}$.

*Step 5.* Calculate the set of the maximum values of the minimum length curve, and the number of the maximum values is denoted as *N*. These points are used as the segmentation points of the spectrum segmentation interval.

*Step 6***.** Establish a band-pass filter based on wavelet transform on the spectrum segmentation interval.

*Step 7.* Reconstruct the signal in order to obtain the IMFs component.

*Step 8*. The power spectrum analysis is performed on the IMFs component in order to extract the fault feature frequency.

### 4.3. Experimental Environment and Results

#### 4.3.1. Experimental Environment and Data

**Figure 4:**
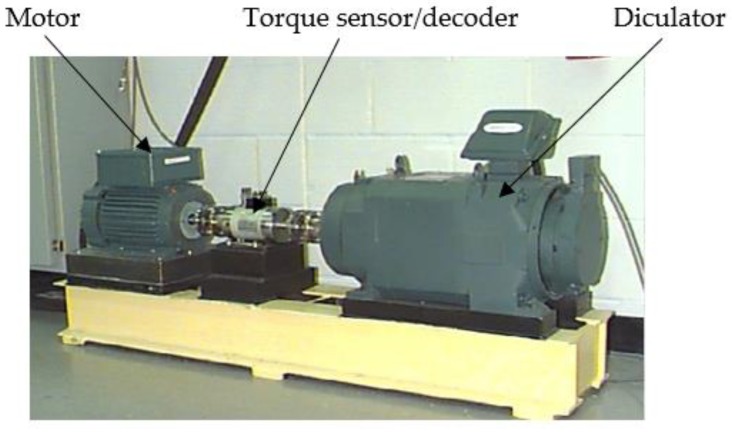
The experimental platform.

The experiment vibration data comes from Bearing Data Center of Case Western Reserve University [34]. The experimental platform is shown in Figure 4. The test stand consists of a 2 hp motor, a torque transducer/encoder, a dynamometer, and control electronics. The experiment uses 6205-2RS JEM SKF deep groove ball bearings. The motor is coupled to the dynamometer and torque sensor through self-aligned coupling. The test bearings support the motor shaft. Single point faults were introduced to the test bearings using electro-discharge machining with fault diameters of 7, 14, 21, 28 and 40 mils (1 mil = 0.001 inches). The data source are the accelerometers on the motor housing of the motor drive end. The vibration signals have a rotational speed of 1797 r/min. Vibration data was collected using accelerometers, which were attached to the housing with magnetic bases. Accelerometers were placed at the 12 o’clock position at both the drive end and fan end of the motor housing. In order to quantify this effect, experiments were conducted for both fan and drive end bearings with outer raceway faults located at 3 o’clock (directly in the load zone), at 6 o’clock (orthogonal to the load zone), and at 12 o’clock. Vibration signals were collected using a 16 channel DAT recorder, and were post processed in a Matlab environment. The vibration signal sampling frequency of the motor bearing is 12,800 Hz, and the duration of each vibration signal is 10 s. Divide the original vibration signals into multiple samples, each with a data sample length of 2048 points. According to the theoretical calculation formula of the fault characteristic frequency, the fault frequency of the outer race, inner race and rolling element of the motor bearing is obtained [35]. The experiment environment is described: Intel Core I5 2450, 4 GB RAM, Win 7 and Matlab 2014b. The fault diameter (14 mils) is selected in here, and the calculated results are shown in Table 1.

#### 4.3.2. Experimental Results

**Figure 5:**
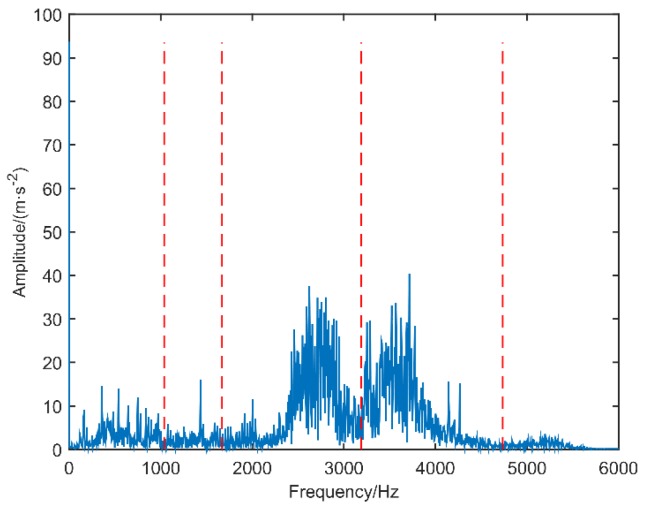
The spectrum segmentation result of motor bearing inner race.

**Figure 6:**
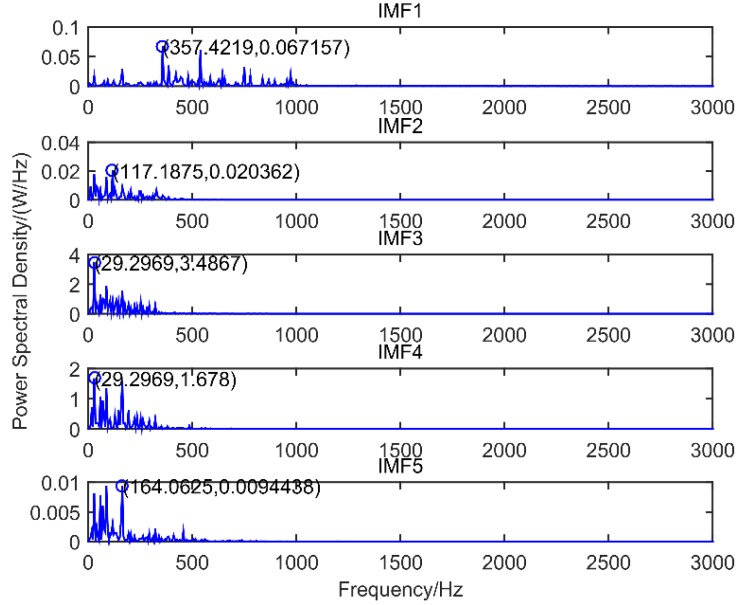
The power spectrum of motor bearing inner race.

The inner race fault vibration signal of motor bearing is selected to verify the effectiveness of the improved scale space representation method. The spectrum segmentation and power spectrum of the inner race vibration signal of the motor bearing are shown in Figure 5 and Figure 6, respectively. The power spectrum results of scale space representation (SSR) and modified scale space representation based on maximum-minimum length curve (MSSR) are compared in Table 2.

As can be seen from Figure 5 and Figure 6 and Table 2, the scale space method is used to segment the inner race vibration signal of motor bearing, and the number of spectrum segmentation intervals is N = 8. According to the power spectrum of the IMFs component, the maximum frequency value of the extracted IMF3, IMF5, IMF6 and IMF7 is 29.30 Hz, the fault feature frequency value of the extracted IMF1 and IMF8 is 164.06 Hz. The modified scale space representation based on maximum-minimum length curve (MSSR) is used to spectrally segment the inner race vibration signal of motor bearing, and the number of spectrum segmentation intervals is N = 5.

According to the power spectrum of the IMFs component, the maximum frequency value of the extracted IMF3 and IMF4 is 29.30 Hz, the maximum frequency value of the extracted IMF1 is 357.42 Hz, the maximum frequency value of the extracted IMF2 is 117.19 Hz, and the maximum frequency value of the extracted IMF5 is 164.06 Hz. 29.30 Hz is the rotation frequency, 117.19 Hz is the quadruple frequency of rotation frequency, 357.42 Hz is the 12 times frequency of rotation frequency, and 164.06 Hz is the fault feature frequency. Therefore, the modified scale space representation based on maximum-minimum length curve reduces the obtained IMFs components of vibration signal from 8 to 5, which reduces the number of adaptive spectrum segmentation and the redundancy of the same frequency, effectively reduces the same modulation of the obtained IMFs and accurately extracts the rotation frequency and the fault feature frequency.

## 5. Experimental Comparison and Analysis

### 5.1. Comparison and Analysis of Improved Scale Space Representation

The EWT methods based on scale space representation, improved scale space representation and LocalMaxmin method are used to decompose the vibration signals of inner race, outer race and rolling ball of the motor bearing. The IMFs components are processed by Hilbert transform, and the feature frequency of each IMF component is extracted by power spectrum.

#### 5.1.1. Result Comparison and Analysis of Inner Race Vibration Signal

The EWT methods based on scale space representation (SSR), modified scale space representation (MSSR) and LocalMaxm in method are used to decompose the inner race vibration signals of the motor bearing. The IMFs components are processed by Hilbert transform and the fault features frequency of IMFs are extracted from the power spectrum. Experimental results of the inner race of motor bearing are shown in Table 3.

As can be seen from Table 3, the scale space method is used to spectrally segment the inner race vibration signals of the motor bearings, and the number of spectrum segmentation intervals is N = 7. According to the power spectrum of the IMFs components, the maximum frequency value of the extracted IMF1 and IMF3 is 58.59 Hz, the maximum frequency value of the extracted IMF2, IMF4, IMF5 and IMF7 is 164.05 Hz, and the maximum frequency value of the extracted IMF6 is 70.31 Hz. The modified scale space method is used to spectrally segment the inner race vibration signal of motor bearing, and the number of spectrum segmentation intervals is N = 4. According to the power spectrum of the IMFs components, the maximum frequency value of the extracted IMF1 is 58.59 Hz, and the maximum frequency value of the extracted IMF2, IMF3 and IMF4 is 164.05 Hz. In order to compare with the modified scale space method, the number of spectrum segmentation intervals of the LocalMaxmin method is set as N = 4. According to the power spectrum of the IMFs components, the maximum frequency value of the extracted IMF1 and IMF4 is 58.59 Hz, and the maximum frequency value of the extracted IMF2 and IMF3 is 164.06 Hz.

From the above analysis, it can be seen that the modified scale space representation based on maximum-minimum length curve reduces the obtained IMFs components of inner race vibration signal of motor bearing from 8 to 5. The modified scale space method reduces the occurrence of false components, reduces the redundancy of the repetition frequency, and can accurately extract the inner race fault feature frequency of motor bearing, the rotation frequency and frequency doubling.

#### 5.1.2. Result Comparison and Analysis of Outer Race Vibration Signal

The EWT methods based on scale space representation (SSR), modified scale space representation (MSSR) and LocalMaxmin method are used to decompose the outer race vibration signals of the motor bearing. The IMFs components are processed by Hilbert transform and the fault features frequency of IMFs are extracted by power spectrum. Experimental results of the outer race of motor bearing are shown in Table 4.

As can be seen from Table 4, the scale space method is used to spectrally segment the outer race vibration signals of motor bearings, and the number of spectrum segmentation intervals is N = 8. According to the power spectrum of the IMF components, the maximum frequency value of the extracted IMF5 and IMF8 is 105.47 Hz, and the maximum frequency values of the remaining IMFs are 11.71, 152.34, 29.30, 52.73, 58.59 and 117.19 Hz, respectively. 11.71 Hz is the 1/9 of the fault feature frequency, 105.47 Hz is the fault feature frequency, 29.30 Hz, 58.59 and 117.18 Hz are the rotation frequency, the rotation frequency doubling and the quadruple rotation frequency respectively. 152.34 Hz and 52.73 Hz are neither multiples of the rotation frequency or the frequency doubling nor the fault feature frequency. From the power spectrum analysis, IMF5 and IMF8 have the same modulation information among the eight obtained IMFs components. The modified scale space method is used to spectrally segment the outer race vibration signal of motor bearing, and the number of spectrum segmentation intervals is N = 4. The maximum frequency value of the extracted IMF1 is 152.34 Hz, the maximum frequency value of the extracted IMF2 is 29.30 Hz, the maximum frequency value of the extracted IMF3 is 105.47 Hz, and the maximum frequency value of the extracted IMF4 is 58.59 Hz. It can be seen that 152.34 Hz is neither the rotation frequency or the frequency doubling nor the fault feature frequency. In order to compare with the modified scale space method, the number of spectrum segmentation of the LocalMaxmin method is set to N = 4. It can be seen from Table 4 that the maximum frequency value of the extracted IMF1 is 87.89 Hz, the maximum frequency value of the extracted IMF2 is 17.58 Hz, the maximum frequency value of the extracted IMF3 is 11.72 Hz, and the maximum frequency value of the extracted IMF4 is 46.88 Hz. From Table 4, it can be seen that 17.57 and 11.71 Hz are 1/6 and 1/9 of the fault feature frequencies, respectively, and 87.89 Hz is the difference between the fault feature frequency and the rotation frequency. 46.88 Hz is neither the rotation frequency or the frequency doubling nor the fault feature frequency.

From the above analysis, it can be seen that that the modified scale space representation based on maximum-minimum length curve reduces the obtained IMFs components of outer race vibration signal of motor bearing from 8 to 4. The modified scale space method reduces the occurrence of false components, reduces the redundancy of the repetition frequency, and can accurately extract the outer race fault feature frequency, the rotation frequency and the frequency doubling of motor bearing. Compared with the LocalMaxmin method, the LocalMaxmin method cannot accurately extract the outer race fault feature frequency, the rotation frequency, and then frequency doubling of motor bearing. Therefore, the effectiveness of the modified scale space method is verified by outer race vibration signal of motor bearing.

#### 5.1.3. Result Comparison and Analysis of Roller Ball Vibration Signal

The EWT methods based on scale space representation (SSR), modified scale space representation (MSSR) and LocalMaxmin method are used to decompose the roller ball vibration signals of the motor bearing. The IMFs components are processed by Hilbert transform and the fault features frequency of IMFs are extracted by power spectrum. Experimental results of the roller ball of motor bearing are shown in Table 5.

As can be seen from Table 5, the scale space method is used to spectrally segment the vibration signals of the roller balls of the motor bearings, and the number of spectrum segmentation intervals is N = 10. According to the power spectrum of the IMFs components, the maximum frequency value of the extracted IMF3, IMF6, IMF8 and IMF10 is 58.59 Hz, the maximum frequency value extracted by IMF5, IMF7 and IMF9 is 29.30 Hz, the maximum frequency value of the extracted IMF1 is 146.48 Hz and the maximum frequency of the extracted IMF2 is 11.72 Hz, and the maximum frequency value of the extracted IMF4 is 140.63 Hz. 11.71 Hz is 1/12 of the fault feature frequency of the roller ball, 140.63 Hz is the fault feature frequency of the roller ball, and 29.30 Hz and 58.59 Hz are the rotation frequency and the frequency doubling respectively.146.48 Hz is neither the rotation frequency or the frequency doubling nor the fault feature frequency. The modified scale space method is used to spectrally segment the vibration signal of the roller ball of the motor bearing, and the number of spectrum segmentation intervals is N = 6. The maximum frequency value of the extracted IMF1 is 117.19 Hz, the maximum frequency value of the extracted IMF2 is 140.63 Hz, the maximum frequency value of the extracted IMF3 is 29.30 Hz, the maximum frequency value of the extracted IMF4 is 222.66 Hz and the maximum frequency value of the extracted IMF5 and IMF6 is 58.59 Hz. 117.18 Hz is the quadruple frequency of the rotation frequency, and 222.65 Hz is the difference between the double fault feature frequency and the double rotation frequency. In order to compare with the modified scale space method, the number of spectrum segmentation of the LocalMaxmin method is set as N = 6. It can be seen from Table 5 that the maximum frequency value of the extracted IMF1 is 117.19 Hz, the maximum frequency value of the extracted IMF2 is 111.33 Hz, the maximum frequency value of the extracted IMF3 is 29.30 Hz, and the maximum frequency value of the extracted IMF4 is 17.58 Hz, the maximum frequency value of the extracted IMF5 is 23.44 Hz, and the maximum frequency value of the extracted IMF6 is 76.17 Hz. 111.33 Hz is the difference between the fault feature frequency and the rotation frequency.17.58 Hz, 23.44 Hz and 76.17 Hz are neither the rotation frequency or the frequency doubling, nor the fault feature frequency. The LocalMaxmin method can only extract four times of the rotation frequency.

From the above analysis, it can be seen that that the modified scale space representation based on maximum-minimum length curve reduces the obtained IMFs components of roller ball race vibration signals of motor bearings from 10 to 6. The modified scale space method reduces the occurrence of false components, reduces the redundancy of the repetition frequency, and can accurately extract the outer race fault feature frequency, the rotation frequency and the frequency doubling of motor bearing. Compared with the LocalMaxmin method, the LocalMaxmin method can only extract four times of the rotation frequency, and is impossible to accurately extract the roller ball fault feature frequency of the motor bearing, the rotation frequency and the frequency doubling of motor bearing. Therefore, the effectiveness of the modified scale space method is verified by outer race vibration signal of motor bearing.

### 5.2. Comparison and Analysis of the MSCEWT Method with EMD and EEMD Methods

In order to verify the effectiveness of the MSCEWT method for vibration signal analysis, the EMD [36] and EEMD [37] methods are selected to compare with MSCEWT method. The vibration signals of inner race, outer race and roller ball of motor bearing are also selected in here.

#### 5.2.1. Result Comparison and Analysis of Inner Race Fault

The proposed MSCEWT method, EMD and EEMD methods are used to analyze the inner race vibration signal of motor bearing. The experimental results are shown in Table 6.

As can be seen from Table 6, the MSCEWT method decomposes the inner race vibration signal to obtain four modes, and the maximum inner race fault feature frequency is 164.06 Hz, the rotation frequency doubling is 58.59 Hz. The EMD method decomposes the inner race vibration signal to obtain nine modes, and the maximum inner race fault feature frequency is 164.06 Hz, and the rests are fractional multiples and irrelevant components of the fault feature frequency. The EEMD method decomposes the inner race vibration signal to obtain 11 modes, and the maximum inner race fault feature frequency is 164.06 Hz, the rotation frequency doubling is 58.59 Hz, but the frequency of maximum repetition rate of the IMFs is too much, and the over-decomposition phenomenon is serious. Compared with EMD and EEMD, the MSCEWT method can not only effectively extract the inner race fault feature frequency of motor bearing, the rotation frequency and the frequency doubling, but also reduces the generation of unrelated components and effectively reduce the number of modes.

#### 5.2.2. Result Comparison and Analysis of Outer Race Fault

The proposed MSCEWT method, EMD and EEMD methods are used to analyze the outer race vibration signal of motor bearing. The experimental results are shown in Table 7.

As can be seen from Table 7, the MSCEWT method decomposes the outer race vibration signal to obtain4 modes, and the maximum outer race fault feature frequency is 105.47 Hz, the rotation frequency is 29.30 Hz, and the double rotation frequency is 58.59 Hz. 152.34 Hz is the difference between twice the fault feature frequency and the double rotation frequency. The EMD method decomposes the outer race vibration signal to obtain nine modes, and the maximum outer race fault feature frequency 105.47 Hz, and the rests are fractional multiples and irrelevant components of the fault feature frequency. The EEMD method decomposes the outer race vibration signal to obtain 11 modes, and the obtained frequency has fault feature frequency and the rotation frequency doubling. However, the frequency of the maximum repetition rate of the IMF component is too much, and the over-decomposition phenomenon is serious. Compared with EMD and EEMD, the MSCEWT method can not only effectively extract the outer race fault feature frequency of motor bearing, the rotation frequency or the rotation frequency doubling, but also reduces the generation of unrelated components and effectively reduce the number of modes.

#### 5.2.3. Result Comparison and Analysis of Roller Ball Fault

The proposed MSCEWT method, EMD and EEMD methods are used to analyze the roller ball vibration signal of motor bearing. The experimental results are shown in Table 8.

As can be seen from Table 8, the MSCEWT method decomposes the roller ball vibration signal to obtain six modes, and the maximum roller ball fault feature frequency is 140.63 Hz, the rotation frequency 29.30 Hz, and the double rotation frequency is 58.59 Hz. 11.72 Hz is 1/12 of the roller ball fault feature frequency of motor bearing. The EMD method decomposes the roller ball vibration signal to obtain 10 modes, and the rotation frequency of roller ball is 29.30 Hz, and the fault feature frequency of roller ball is not obtained, the rests are the fractional multiple and the irrelevant components of the fault feature frequency. The EEMD method decomposes the roller ball vibration signal to obtain 11 modes, and the obtained rotation frequency of roller ball is 29.30 Hz. The fault feature frequency is not obtained, and the irrelevant component decomposition is excessive. Compared with EMD and EEMD methods, the MSCEWT method can not only effectively extract the fault roller ball feature frequency, the rotation frequency and the rotation frequency doubling of motor bearing, but also reduces the generation of irrelevant components and effectively reduce the number of modes.

In summary, when the proposed MSCEWT method is used to decompose the vibration signal of motor bearing, it can not only reduce the number of the modes, but also can obtain the fault feature frequency, the rotation frequency and the frequency doubling of motor bearing by power spectrum. The EMD and EEMD methods are used to decompose the vibration signals of motor bearings, they obtain more modes. For the inner race and outer race fault vibration signals, the EMD and EEMD methods can accurately obtain the fault feature frequency, but they cannot obtain the rotation frequency. For the roller ball fault vibration signal, the EMD and EEMD methods can only obtain the rotation frequency, but they cannot obtain the fault feature frequency. Compared with EMD and EEMD methods, the proposed MSCEWT method can not only effectively extract the fault feature frequency, the rotation frequency and the frequency doubling of the inner race, outer race and roller ball of motor bearing, but it also reduces the generation of unrelated components and effectively reduce the number of IMFs components. Therefore, the MSCEWT method is effective decomposition method for vibration signal of rotating machinery.

## 6. Conclusions

Empirical wavelet transform (EWT) is a novel adaptive signal decomposition method, but in actual applications, the main shortcoming of the EWT method is that its Fourier segmentation is strongly dependent on the local maxima of the amplitudes of the Fourier spectrum of the signal. If the Fourier spectrum of the signal is complicated and overwhelmed by strong noises and other strong vibration signals, the Fourier segmentation is not always reliable and effective. In this paper, an enhanced empirical wavelet transform (MSCEWT) based on a maximum-minimum length curve method is proposed for fault diagnosis of motor bearings. Firstly, an improved scale space representation method based on maximum-minimum length curve is proposed to enhance the EWT method. The maximum-minimum length curve method transforms the spectrum of the original vibration signal to scale space in order to obtain a set of minimum length curves, and find the maximum length curve value in the set of the minimum length curve values for obtaining the number of the spectrum decomposition intervals. Then the MSCEWT method decomposes the vibration signal into a series of IMFs. Finally, the frequency of each component is extracted by power spectrum and compared with the theoretical values of motor bearing fault feature frequencies in order to determine and obtain the fault diagnosis results. Actual motor bearing vibration signals are selected and the EMD and EEMD methods are selected for comparative analysis. It can be seen from the experimental results that the MSCEWT method has fewer decomposition modes, and the fault feature frequency, the rotation frequency and the rotation frequency doubling can be accurately extracted. This study thus provides a new method for fault diagnosis of rotating machinery.

## Figures and Tables

**Table 1:** Fault feature frequencies.

Inner Race	Outer Race	Ball	Rotation Frequency
162.19 Hz	107.29 Hz	141.08 Hz	29.93 Hz

**Table 2:** Power spectrum results of two methods.

	IMF1	IMF2	IMF3	IMF4	IMF5	IMF6	IMF7	IMF8
SSR	164.06	93.75	29.30	117.19	29.30	29.30	29.30	164.06
MSSR	357.42	117.19	29.30	29.30	164.06			

**Table 3:** The experimental results of inner race of motor bearing.

	IMF1	IMF2	IMF3	IMF4	IMF5	IMF6	IMF7
SSR	58.59	164.06	58.59	164.06	164.06	70.31	164.06
LocalMaxmin	58.59	164.06	164.06	58.59			
MSSR	58.59	164.06	164.06	164.06			

**Table 4:** The experimental results of outer race of motor bearing.

	IMF1	IMF2	IMF3	IMF4	IMF5	IMF6	IMF7	IMF8
SSR	11.71	152.34	29.30	52.73	105.47	58.59	117.19	105.47
LocalMaxmin	87.89	17.58	11.72	46.88				
MSSR	152.34	29.30	105.47	58.59				

**Table 5:** The experimental results of roller ball of motor bearing.

	IMF1	IMF2	IMF3	IMF4	IMF5	IMF6	IMF7	IMF8	IMF9	IMF10
SSR	146.48	11.72	58.59	140.63	29.30	58.59	29.30	58.59	29.30	58.59
LocalMaxmin	117.19	111.33	29.30	17.58	23.44	76.17				
MSSR	117.19	140.63	29.30	222.66	58.59	58.59				

**Table 6:** The experimental results of inner race of the motor bearing.

	IMF1	IMF2	IMF3	IMF4	IMF5	IMF6	IMF7	IMF8	IMF9	IMF10	IMF11
EMD	164.06	164.06	164.06	158.20	17.58	23.44	5.86	5.86	5.86		
EEMD	164.06	164.06	164.06	58.59	41.02	41.02	23.44	23.44	11.72	11.72	5.86
MSCEWT	58.59	164.06	164.06	164.06							

**Table 7:** The experimental results of the outer race of the motor bearing.

	IMF1	IMF2	IMF3	IMF4	IMF5	IMF6	IMF7	IMF8	IMF9	IMF10	IMF11
EMD	105.47	46.88	152.34	5.86	17.58	5.86	11.72	11.72	5.86		
EEMD	105.47	105.47	105.47	58.59	58.59	17.59	17.59	5.86	5.86	5.86	5.86
MSCEWT	152.34	29.30	105.47	58.59							

**Table 8:** The experimental results of roller ball of the motor bearing.

	IMF1	IMF2	IMF3	IMF4	IMF5	IMF6	IMF7	IMF8	IMF9	IMF10	IMF11
EMD	222.66	328.13	11.72	41.02	29.30	11.72	11.72	5.86	5.86	5.86	
EEMD	222.66	222.66	328.13	146.48	169.92	29.30	17.58	11.72	5.86	5.86	5.86
MSCEWT	117.19	140.63	29.30	222.66	58.59	58.59					
